# Supplementary material for: Nanostructured ZnO/Ag Film Prepared by Magnetron Sputtering Method for Fast Response of Ammonia Gas Detection
Source: Molecules. 2020 Apr 20;25(8):1899. doi: 10.3390/molecules25081899 (PMC7221679; doi:10.3390/molecules25081899)
Supplement: Supplementary file 1 [file molecules-25-01899-s001.pdf]

Supporting Information

Nanostructured ZnO/Ag Film Prepared by Magnetron Sputtering Method for Fast Response of Ammonia Gas Detection

Yiran Zheng [1], Min Li [1], Xiaoyan Wen [1], Ho-Pui Ho [2] and Haifei Lu [1,*]

[1] School of Science, Wuhan University of Technology, Wuhan 430070, China; yiranzzzzz@whut.edu.cn (Y.Z.); minli@whut.edu.cn (M.L.); wenxy@whut.edu.cn (X.W.)

[2] Department of Biomedical Engineering, The Chinese University of Hong Kong, Shatin, Hong Kong, China; aaron.ho@cuhk.edu.hk

* Correspondence: haifeilv@whut.edu.cn

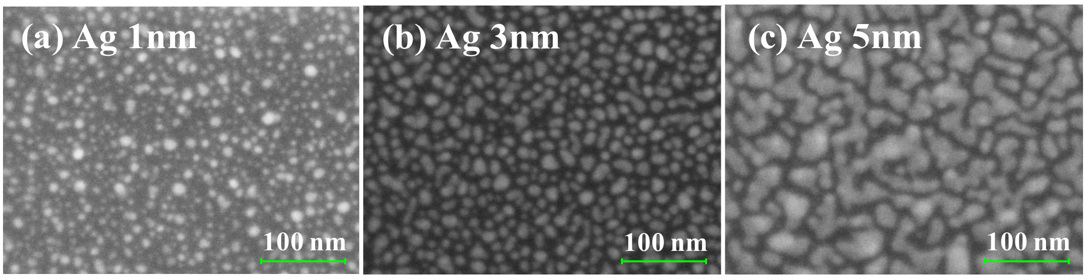

Figure S1. The SEM micrograph of Ag layers with **(a)** 1 nm, **(b)** 3 nm, and **(c)** 5 nm.

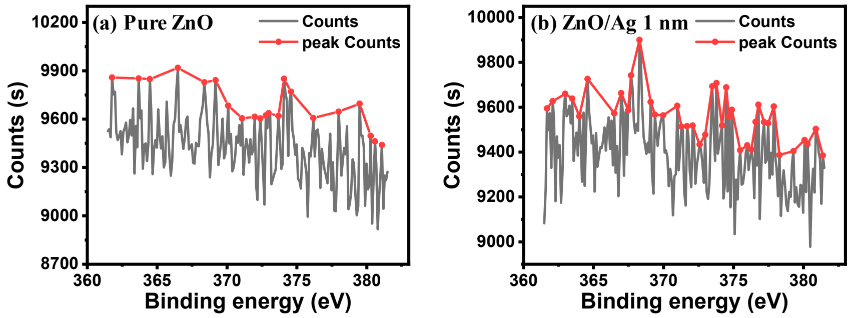

Figure S2. The XPS spectrum of Ag element measured on the pure ZnO and ZnO/Ag (1 nm) film.

Table S1. The parameters and calculations of the average crystallite size of the ZnO crystal using Scherrer equation.

	K	λ/nm	β	θ	D/nm
**Pure ZnO**	0.943	0.154	0.0098298	0.296	15.450
**ZnO/Ag (1 nm)**	0.943	0.154	0.0051156	0.301	29.734
**ZnO/Ag (3 nm)**	0.943	0.154	0.0086982	0.301	17.487
**ZnO/Ag (5 nm)**	0.943	0.154	0.0069072	0.301	22.021

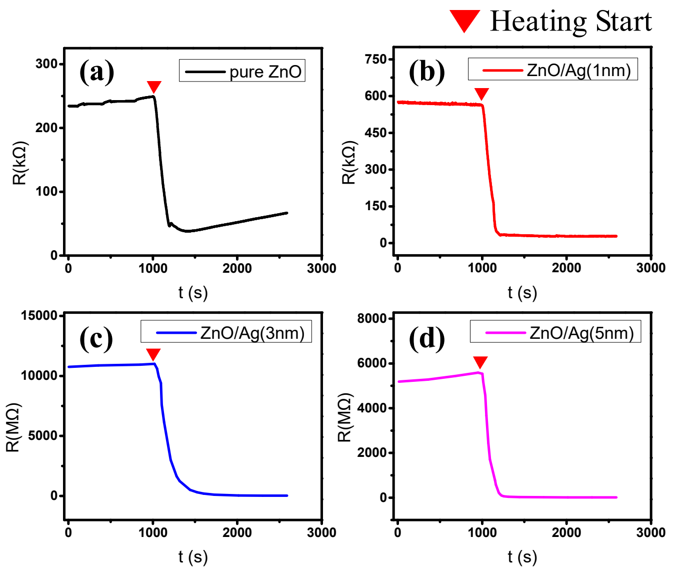

Figure S3. The resistance of ZnO films with different Ag thickness before and after heating.

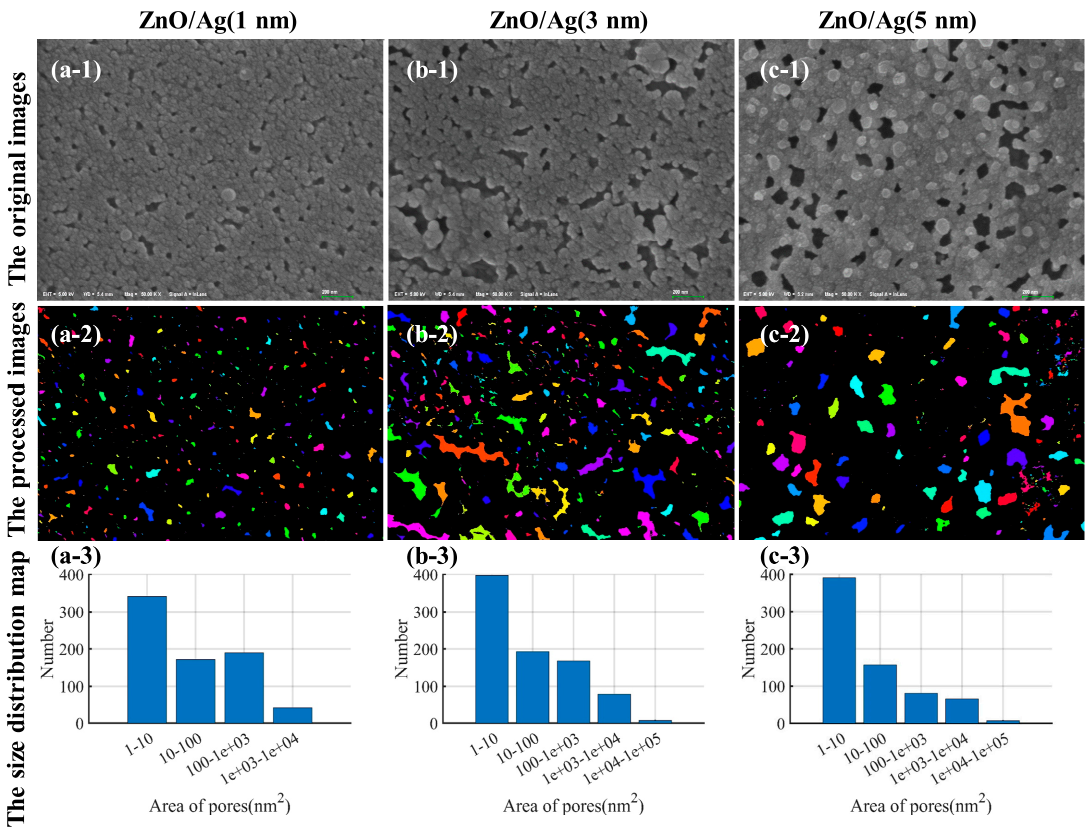

Figure S4. The statistics analysis of ZnO films with **(a)** 1 nm, **(b)** 3 nm and **(c)** 5 nm Ag: **(1)** SEM images of the three nanostructured film and **(2)** corresponding colored figures of the nanopores in the films. **(3)** The distribution diagrams of pores on the three ZnO/Ag films.

Table S2. The statistics summary of area and quantity of nanopores in the three nanostructured films.

	Total Area of Film Surface (nm^2)	Quantity of all Pores	Total Area of Pore Entrance (nm^2)	Area Ratio of Pore Entrance and Film Surface	Total Area of Pore Entrance above 1963.4954 nm (nm^2)
**ZnO/Ag (1 nm)**	3417867.4073	743	143340.1763	0.041938	27368.6753
**ZnO/Ag (3 nm)**	3417867.4073	843	405265.0646	0.11857	289380.5096
**ZnO/Ag (5 nm)**	3417867.4073	605	353010.5060	0.10328	303789.3974

We have calculated the average crystallite size of the ZnO crystal using Scherrer equation $D = K \lambda/(\beta \cos\theta)$, where D is the average crystallite size, K is the shape constant (K = 0.89 when the particles are spherical or K = 0.943 when the particles are cubic), λ is the X-ray wavelength(0.154 nm), β is the half-width of the full maximum (HWFM) and θ is the angle value. The average crystallite sizes of the ZnO/Ag (1, 3, 5) are 29.73 nm, 17.49 nm, 22.02 nm, respectively. Scherrer equation applies only to unstressed films. Thus, the average crystallite size of pure ZnO films is 15.45 nm for reference only.

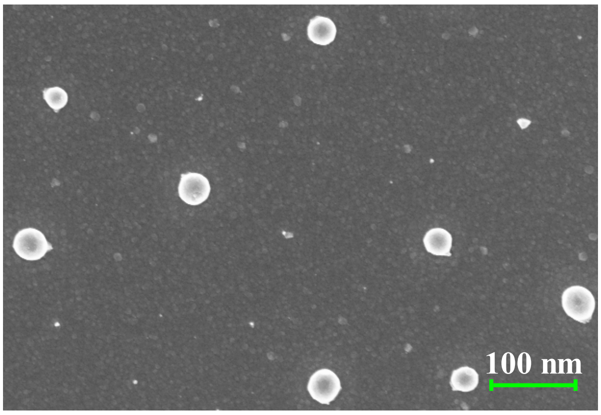

Figure S5. The SEM micrograph of up-Ag 3 nm/ZnO films.

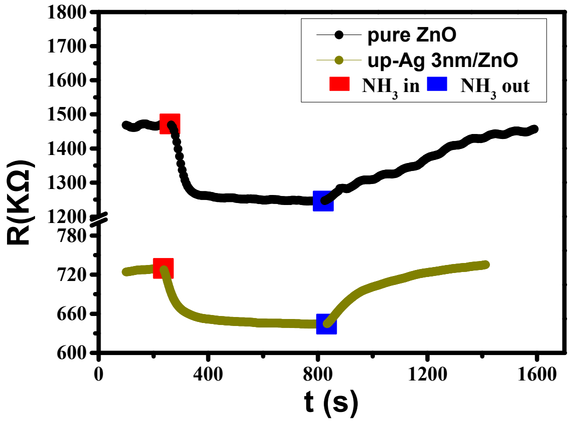

Figure S6. Sensing performance of ZnO films with 5 nm Ag exposed to 300 ppm NH3, comparing to pure ZnO.

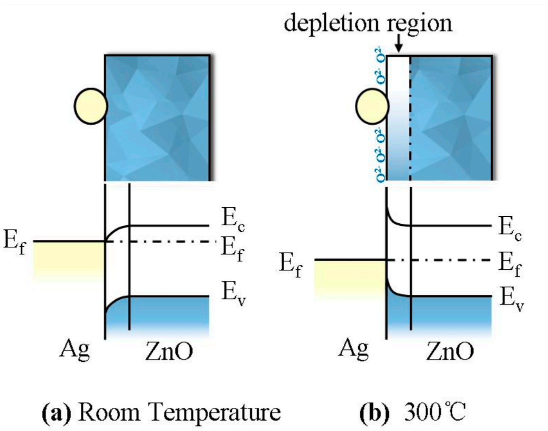

Figure S7. Schematic of the band diagram of silver and ZnO nanograins under **(a)** room temperature and **(b)** 300 °C.
